# Outpatient overstitch repair of perforation following endoscopic submucosal dissection: a novel, cost-effective approach

**DOI:** 10.1055/a-2787-1628

**Published:** 2026-02-24

**Authors:** Hugo Gonçalo Guedes, Julia Schettini Veloso, Juliana de Meneses, Julio Cesar de Soares Veloso

**Affiliations:** 1 42522 Departamento de Endoscopia Digestiva, Hospital Sírio-Libanês, Brasília, Brazil; 2 468920 Curso de Medicina, UniCEUB Faculdade de Ciências da Educação e Saúde, Brasília, Brazil; 3 283325 Centro de Endoscopia, Hospital de Base do Distrito Federal, Brasília, Brazil; 4 200242 Instituto do Aparelho Digestivo de Brasília (IAD), Hospital Anchieta, Brasília, Brazil

The OverStitch NXT single-channel suturing system is a novel endoscopic approach for outpatient closure of intra-procedural perforations. Compared to its double-channel predecessor, this device can be mounted on conventional endoscopes and presents increased flexibility and maneuverability. Thus, it is a technically feasible and safe method for endoscopic suturing.

**Fig. 1:**
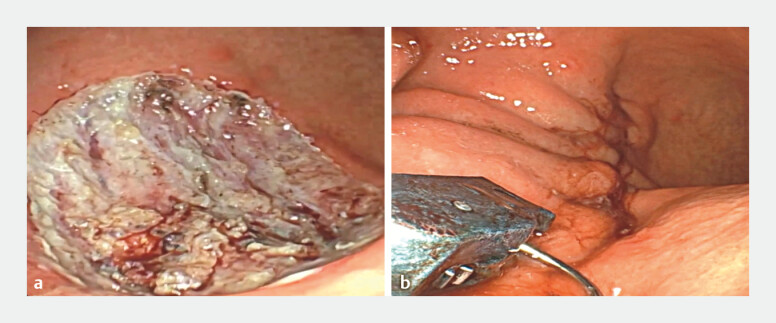
Comparison of the endoscopic view of **a** an endoscopic submucosal dissection (ESD) lesion with intra-procedural perforations and the **b** ESD lesion after endoscopic overstitch suturing.

We report here a case of an 80-year-old patient, with high cardiac risk, with intra-procedural endoscopic submucosal dissection (ESD) complications, after full-thickness perforation and pneumoperitoneum occurred, following endoscopic dissection of a 2 cm well-differentiated gastric adenocarcinoma ( Video 1 ). The pneumoperitoneum was drained percutaneously, and after the completion of the dissection, the mucosal defect was closed using the OverStitch NXT single-channel suturing system ( Fig. 1 ). The patient recovered fully without complications and was discharged within 24 hours.

Endoscopic aspect of full thickness endosuture after ESDʼs perforation.Video 1

Intraoperative perforations during ESD occur in approximately 2.3% of cases, and endoscopic closure should be considered first. When endoscopic closure is successfully achieved, patients can be managed conservatively with fasting, installation of nasogastric tube, and antimicrobial therapy, reducing global hospital costs and the need for surgical interventions 1 .

The use of standard clips have important limitations, as they typically grasp only the mucosa, may create submucosal dead space prone to wound dehiscence, and are often technically challenging in larger defects, requiring multiple applications that increase procedural time and cost 2 3 . In contrast, endoscopic suturing provides reliable tissue approximation and has been associated with early, same-day discharge in a substantial proportion of patients, suggesting that the reduced length of stay and avoidance of prolonged hospitalization may help offset device-related costs, while preserving patient safety and comfort 3 4 . Therefore, novel suturing devices, such as the OverStitch NXT single-channel system, present minimally invasive, cost-effective and efficient closure of defects in therapeutic endoscopic procedures like ESD.

Endoscopy_UCTN_Code_TTT_1AO_2AO
